# Combining lipiodol-based indirect radiography and intraoperative methylene blue for sentinel lymph node mapping in canine cutaneous mast cell tumors

**DOI:** 10.1007/s11259-026-11369-z

**Published:** 2026-06-26

**Authors:** Jéssica Francielle Camargo, Simone Passos Bianchi, Haiumy Garcia Cardozo, Caroline Soares Ferrari, Gabriela Foppa, Rodrigo Santos dos Horta, Priscila Beatriz Silva da Serpa, Stella Faria de Valle

**Affiliations:** 1 Veterinary Teaching Hospital, Universidade Federal do Rio Grande do Sul (UFRGS), Porto Alegre, RS Brazil; 2 Department of Veterinary Medicine and Surgery, Universidade Federal de Minas Gerais (UFMG), Belo Horizonte, MG Brazil; 3 Department of Population Medicine and Diagnostic Sciences, Cornell University, Ithaca, NY USA; 4 Departament of Veterinary Clinical Pathology, Universidade Federal do Rio Grande do Sul (UFRGS), Av. Bento, Porto Alegre, RS 9090, CEP 91540-000 Brazil

**Keywords:** Mast cell tumor, Lymphatic Metastasis, Lymphadenectomy, Oncology

## Abstract

Canine cutaneous mast cell tumor (cMCT) is one of the most common skin neoplasms in dogs, with lymph nodes representing the primary route of metastasis. Accurate sentinel lymph node (SLN) identification is therefore essential for staging and treatment planning. This study evaluated indirect lipiodol lymphography (IL) and intraoperative methylene blue (MB) mapping in 21 dogs, including 26 tumors and 38 excised lymph nodes, to assess SLN detection and its association with tumor characteristics and nodal status. MB identified 34 SLNs, whereas IL detected 29. The two techniques were concordant for 21 SLNs. In five tumors in which IL failed to identify a SLN, MB subsequently detected six axillary SLNs. Concordance with the expected regional lymph node (RLN) was higher for MB (76.9%) than for IL (61.5%) and increased to 84.6% when both techniques were combined, highlighting their complementary value for SLN mapping. MB detected additional axillary nodes, whereas IL aided in identifying some inguinal nodes. Larger tumors were more frequently associated with incomplete surgical margins and higher nodal histologic grades, indicating an increased risk of metastasis. Importantly, 19% of tumors drained to at least one SLN outside the expected regional basin, highlighting lymphatic anatomical variability. The absence of IL opacification did not exclude clinically relevant nodes, as MB identified all such cases, including HN0–HN2 nodes. Although MB stained some non-metastatic nodes, it provided useful intraoperative guidance. Adverse events were uncommon, with two transient reactions after IL and none after MB, and postoperative morbidity was low (13.2%; 5/38). Overall, MB offered higher detection rates, while IL improved anatomical delineation. Combining lipiodol-based indirect radiography and intraoperative MB staining offers a highly effective, complementary approach for identifying the SLN in dogs with cMCTs.

## Introduction

Canine cutaneous mast cell tumors (cMCTs) are among the most common skin neoplasms in dogs, accounting for approximately 16% to 21% of all diagnosed cutaneous tumors in these species (Strefezzi et al. 2009). The sentinel lymph node (SLN) is the first lymph node to receive metastatic cells from a solid tumor and is therefore usually the first site where tumor cells migrate after leaving the primary tumor. However, the SLN may be located in anatomically atypical sites and may not be correspond to the expected regional lymph node (RLN) (Gould et al. 1960; Christiansen and Detmar 2011; Worley 2014). In human oncology, it is well documented that selective lymphadenectomy can result in less pain and reduced postoperative morbidity (Veronesi et al. 2003). In dogs with cMCT, the removal of metastatic SLNs has been associated with improved prognostic outcome (Sabattini et al. 2021; Chalfon et al. 2022).

Over recent decades, methods for identifying nodal metastasis in canine mast cell tumors (MCTs) have improved, with several techniques described in the literature for both preoperative and intraoperative detection (Brissot and Edery 2017; Collivignarelli et al. 2021; Zanardi et al. 2025). Lymphatic fluid is colorless, which makes intraoperatively identification of lymphatic vessels difficult without the use of dyes (Suami 2017).

A previous study demonstrated the efficacy of indirect lymphangiography with lipiodol in mapping the SLN in dogs with cMCT, including evidence that multiple lymph nodes may be involved and that the lymphatic drainage pattern vary among tumors (De Bonis et al. 2022). However, one limitation described was the lack of intraoperative confirmation using dyes, making it impossible to verify whether the lymph nodes identified radiographically corresponded to those removed during surgery. To address this gap, we aimed to quantify how preoperative iodized-oil lymphangiography (Lipiodol; IL) and intraoperative methylene blue (MB) mapping complement each other in identifying SLNs in cMCT. We hypothesized that MB could be a cost-effective adjunct to preoperative Lipiodol mapping, improving real-time lymph node identification without increasing morbidity.

## Material and methods

### Study design and case selection

This prospective study was approved by the Ethics Committee on Animal Use from Universidade Federal do Rio Grande do Sul (UFRGS), under protocol number 43,437. Written informed consent was obtained from all owners before enrollment. All clinical evaluations and therapeutic procedures followed standard-of-care practices and were not influenced by study participation. No additional interventions or discomfort were imposed for research purposes. Exclusions criteria included non-resectable tumors; prior surgery or scar tissue near the tumor or the expected SLN basin; neoadjuvant, ongoing, or previous chemotherapy; hypersensitivity to contrast agents; renal disease; concurrent tumors; or a history of neoplasia within the previous five years.

A total of 21 client-owned dogs diagnosed with cMCTs were prospectively enrolled at the Veterinary Teaching Hospital of UFRGS between August 2023 and February 2025. Dogs of any breed, sex, or age were eligible, provided they met the predefined inclusion and exclusion criteria.

All dogs underwent a standardized staging protocol, which included oncological clinical evaluation, comprehensive physical examination, fine-needle aspiration biopsy (FNA) with cytological assessment for tumor triage, complete blood count, serum biochemical analysis, abdominal ultrasonography, and three-view thoracic radiography.

## Indirect lymphography

For dogs eligible and selected for surgery, SLN identification was performed using iodized oil (IO; Lipiodol Ultra-Fluid, iodized ethyl-esters of the fatty acids of poppy seed oil, iodine 480 mg/mL; Guerbet Ltda, Brazil). The contrast agent was injected intradermally using a 25 G needle attached to a 1 mL syringe in the four quadrants surrounding the tumor, approximately 0.5 to 1 cm from the tumor margins. The procedure followed a protocol previously described (Collivignarelli et al. 2021). A volume of 0.4 mL was slowly injected into each quadrant, adjusted according to tumor size, over 1–2 min. After injection, the site was gently massaged to facilitate lymphatic drainage, with particular care taken to avoid intravascular administration of the contrast agent. In certain cases, due to tumor size, promethazine was administered as premedication at a dose of 0.2 mg/kg prior to contrast injection to prevent mast cell degranulation. Animals were closely monitored for 10 min after injection for any signs of adverse reactions.

Three radiographic projections were obtained 24 h after contrast administration to optimize visualization of the SLN, considering the tumor’s anatomical location and the expected lymphatic drainage pathway. The SLN was identified based on these projections. For tumors located in the abdominal region, some dogs were recommended to fast prior to imaging to improve visualization of the contrast-enhanced lymph node.

## Intraoperative SLN mapping with MB and surgical procedure

Patients were referred to the general surgery service and anesthetized according to individualized protocols based on physical status classification. Anesthetic monitoring was performed continuously following institutional guidelines.

Subsequently, 0.5 mL of sterile methylene blue (MB; 5 mg/mL; Renylab, Brazil) was injected intradermally into four peritumoral quadrants using a 3 mL syringe and a 25 G needle. After a 30-minute diffusion period, SLN excision (lymphadenectomy) was performed before the tumor excision to avoid seeding neoplastic cells to the LN sites. All contrasted-enhanced and/or blue-stained SLNs were accessed for lymphadenectomy. SLN localization during surgery was guided primarily by the lymphographic findings obtained after indirect lymphography, whereas MB was used as an adjunctive technique to facilitate intraoperative visualization of lymphatic vessels and SLNs. When SLN mapping could not be achieved due to the absence of both contrast and dye, a non-selective dissection of the RLN was performed, following the anatomical distribution of lymphatic territories previously described in normal dogs (Suami et al. 2013). Lymphadenectomy was performed using sharp and blunt dissection. The blood vessels of the lymph nodes were ligated using 3 − 0 polydioxanone (PDS; Bioline, Brazil) or coagulated using an electrosurgical device.

Tumor excision was then performed with lateral surgical margins of 2 to 3 cm and one fascial plane in depth. However, after margins assessment, it was observed that 2–3 cm margins were insufficient for complete excision in some cases. Therefore, an intraoperative biopsy was performed when necessary. Lateral margins were demarcated using a dermatographic pen (Texta 700; Texta, Brazil). In some cases, intraoperative biopsies were performed, whereas in others, tumor samples were submitted directly for routine histopathological analysis using surgical inks of different colors (yellow, black, and green), following the protocol previously described (Kamstock et al. 2011). The use of surgical ink aimed to assist in sample orientation, facilitating visual identification of margins both macroscopically and microscopically, and highlighting areas of particular concern.

Both excised tumor tissues and lymph nodes were measured and fixed in 10% neutral buffered formalin for subsequent histopathological analysis at the Veterinary Pathology Laboratory of UFRGS.

The diagnosis of cMCT was confirmed histologically according to the Patnaik three-tier grading system (Patnaik et al. 1984) and the Kiupel two-tier grading system (Kiupel et al. 2011). The presence of lymph node metastasis was assessed and classified according to the HN system (Weishaar et al. 2014). Surgical margins were classified as incomplete when neoplastic mast cells were present at the cut edge of the specimen and complete when no neoplastic cells were detected at the margin (histologic tumor-free margin >0 mm).

### Statistical analysis

Age, weight, and tumor size were presented as mean ± SD or median (IQR) after assessment of normality using the Shapiro-Wilk test. Categorical data are presented as count and percentages. Outcomes are described in terms of detection and complication rates, with exact 95% confidence intervals (95% CI). IL and MB were evaluated in the same tumors and compared using McNemar’s test. Agreement is reported as percent agreement and Cohen’s kappa with 95% CI. Associations between categorical variables (e.g., tumor size group and nodal metastasis, margin width and completeness) were analyzed using Fisher’s exact test. Continuous two-group comparisons were assessed using the t-test or the Mann–Whitney U test. Analyses were conducted per tumor; however, one tumor per dog (the largest tumor) was used for the size–metastasis analysis. All tests were two-sided with α = 0.05 and were conducted in R (R Core Team, 2025).

## Results

### Patient demographics and tumor characteristics

Twenty-one dogs completed the study. As some dogs presented with multiple cMCTs, a total of 26 tumors were evaluated, resulting in the excision of 38 lymph nodes. All tumors were histologically classified as cutaneous mast cell tumors. The most prevalent tumor sites were the trunk (11 cases, 42.3%) and thoracic limb (8 cases, 30.8%), followed by the pelvic limb (4 cases, 15.4%), head and neck (2 cases, 7.7%), and perineal region (1 case, 3.8%).

Table 1 summarizes the characteristics of the animals included in the study, as well as their tumor locations. Ten breeds were represented, including eight mixed-breed dogs and two Golden Retrievers; other breeds represented by a single dog included the Pug, Fox Terrier, French Bulldog, Pinscher, Labrador Retriever, Bernese Mountain Dog, and Rottweiler. The median age of the dogs included in this study was 9.5 years (range 5–13 years). Seventeen female and four male dogs were included in this study.

**Table 1:** Demographics and tumor characteristics of 21 dogs with cutaneous mast cell tumors (cMCT) undergoing sentinel lymph node (SLN) mapping using iodized oil (IO) and methylene blue (MB)

Characteristic	Value (%)
Breed	
Mixed Breed	11 (52.4)
Golden Retriever	2 (9.5)
Bernese Mountain Dog	1 (4.8)
Dachshund	1 (4.8)
Fox Terrier	1 (4.8)
French Bulldog	1 (4.8)
Labrador Retriever	1 (4.8)
Pinscher	1 (4.8)
Pug	1 (4.8)
Rottweiler	1 (4.8)
Sex	
Female	17 (81%)
Male	4 (19%)
Bodyweight	18.9 ± 12.7 kg (range, 4.2–47.0 kg)
Age	9.5 ± 2.1 years (range, 5–13 years)
MCT location	
Head/neck	2 (7.7%)
Trunk	11 (42.3%)
Forelimb	8 (30.8%)
Hindlimb	4 (15.4%)
Perineal	1 (3.8%)

Abbreviations: MCT (mast cell tumor)

### Indirect lymphography mapping

**Fig. 1:**
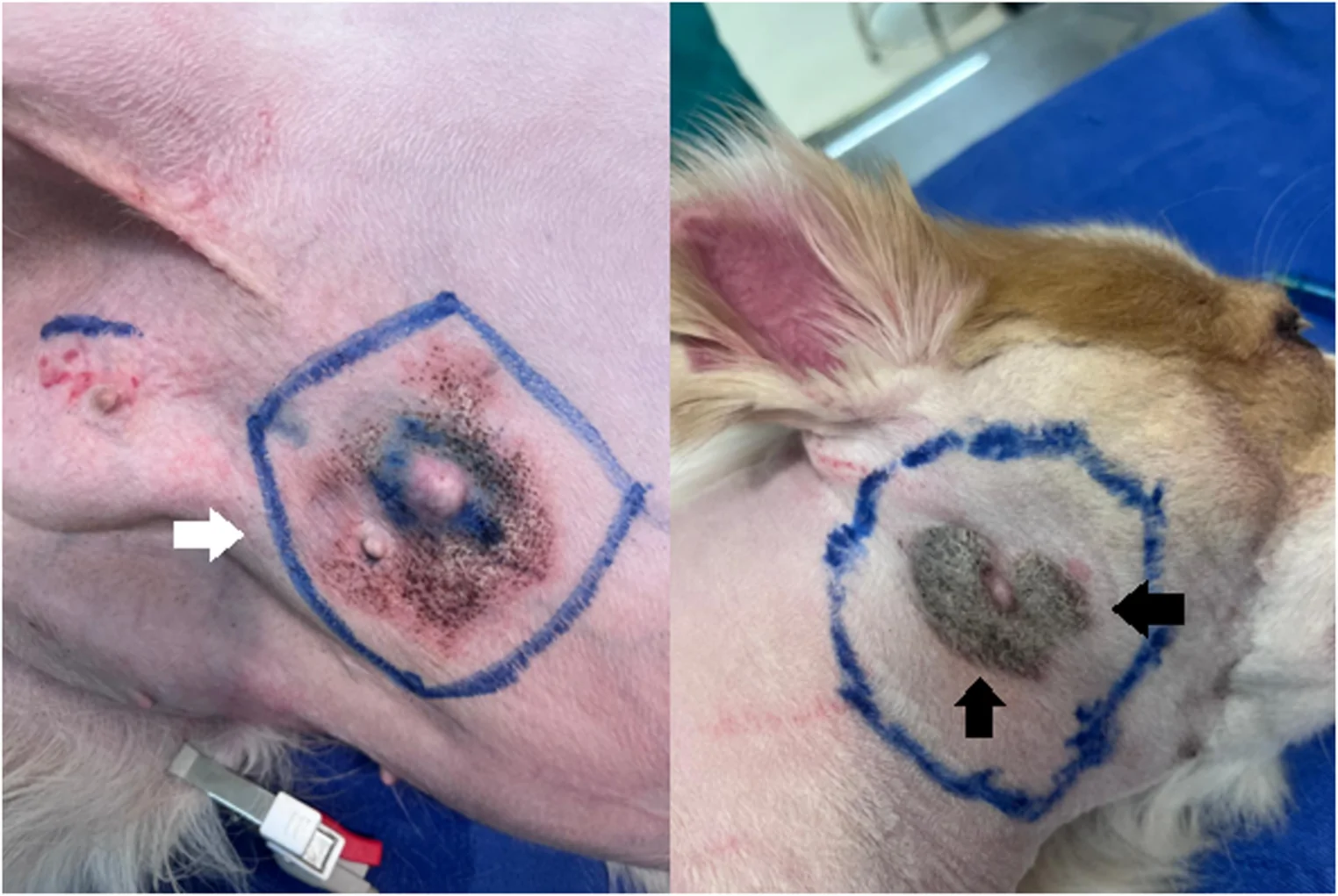
Local reaction was seen 24 hours after peritumoral intradermal injection of iodized oil (Lipiodol). (A) White arrow: injection papule/entry point. (B) Localized gray discoloration; black arrows indicate areas with a necrotic appearance (complatible with superficial epidermal necrosis)

Surgery was generally performed five to seven days after IO injection, according to institutional scheduling logistics. Mild post-injection changes (> 24 h), such as transient hyperemia and occasional nodule enlargement, were observed in some dogs. Three dogs (Fig. 1) developed localized adverse reactions at the site of IO administration, characterized clinically by erythema and swelling. Surgical excision of the affected tissue was performed, and histopathological examination revealed focal lymphoplasmacytic dermatitis. No immediate systemic adverse reactions associated with peritumoral IO injection were observed.

**Fig. 2:**
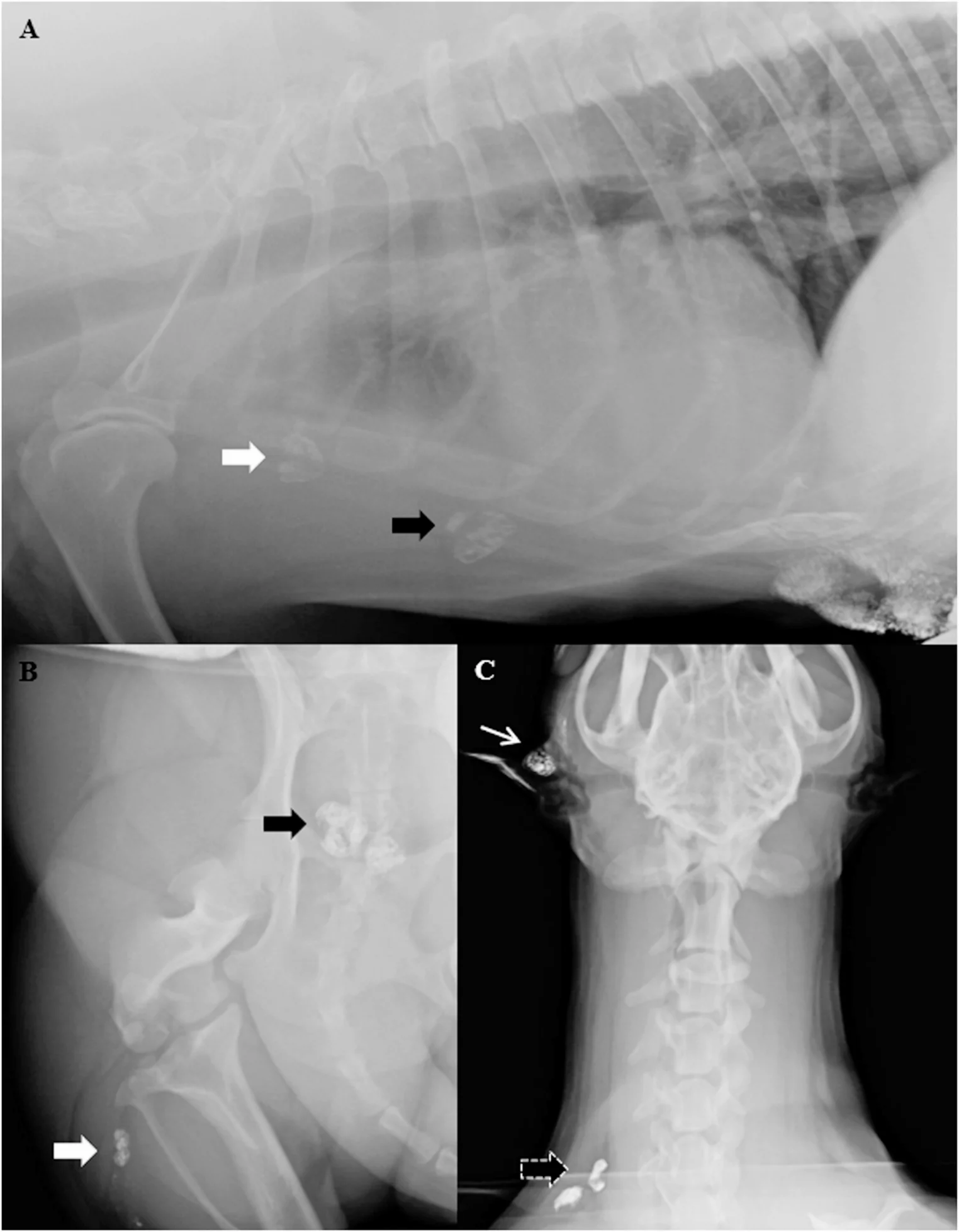
Radiographic lymphography in dogs. (A) Two lymph nodes after IL in the mammary region: black arrowhead, accessory axillary LN; white arrowhead, axillary LN. (B) Three lymph nodes visualized: white arrowhead, popliteal LN; black arrowhead, two superficial inguinal LNs. (C) Two lymph nodes after Lipiodol injection in the ear pinna: dotted black arrowhead, ventral and dorsal superficial cervical LNs

A total of 29 SLNs were identified in 26 tumors using IO. Among the 21 dogs with 26 tumors, lipiodol allowed detection of the SLNs in 16 dogs (21 tumors). In five tumors (19.2%), no contrasted-enhanced SLN was detected, even after repeating the injection. Eight tumors presented more than one SLN. The number of SLN per dog ranged from 0 to 3, with a mean of 1.15 ± 0.78 and a median of 1. Among the tumors with detectable nodes, most had a single SLN (61.9%, *n* = 13), followed by two nodes (33.3%, *n* = 7) and three nodes (4.8%, *n* = 1) (Fig. 2).

### Intraoperative methylene blue colorimetric mapping 

A total of 34 SLNs were detected in 26 tumors using MB. Among the 21 dogs with 26 tumors, MB enabled detection of the SLN in 19 dogs (24 tumors). Dye uptake could only be confirmed during surgical exposure of the lymph nodes. In some dogs, a small amount of dye was visible through the skin; however, it was not possible to determine exactly which nodes were stained. SLN localization was guided by lipiodol lymphography, and during surgery the nodes identified by lipiodol were carefully examined for MB staining. 

**Fig. 3:**
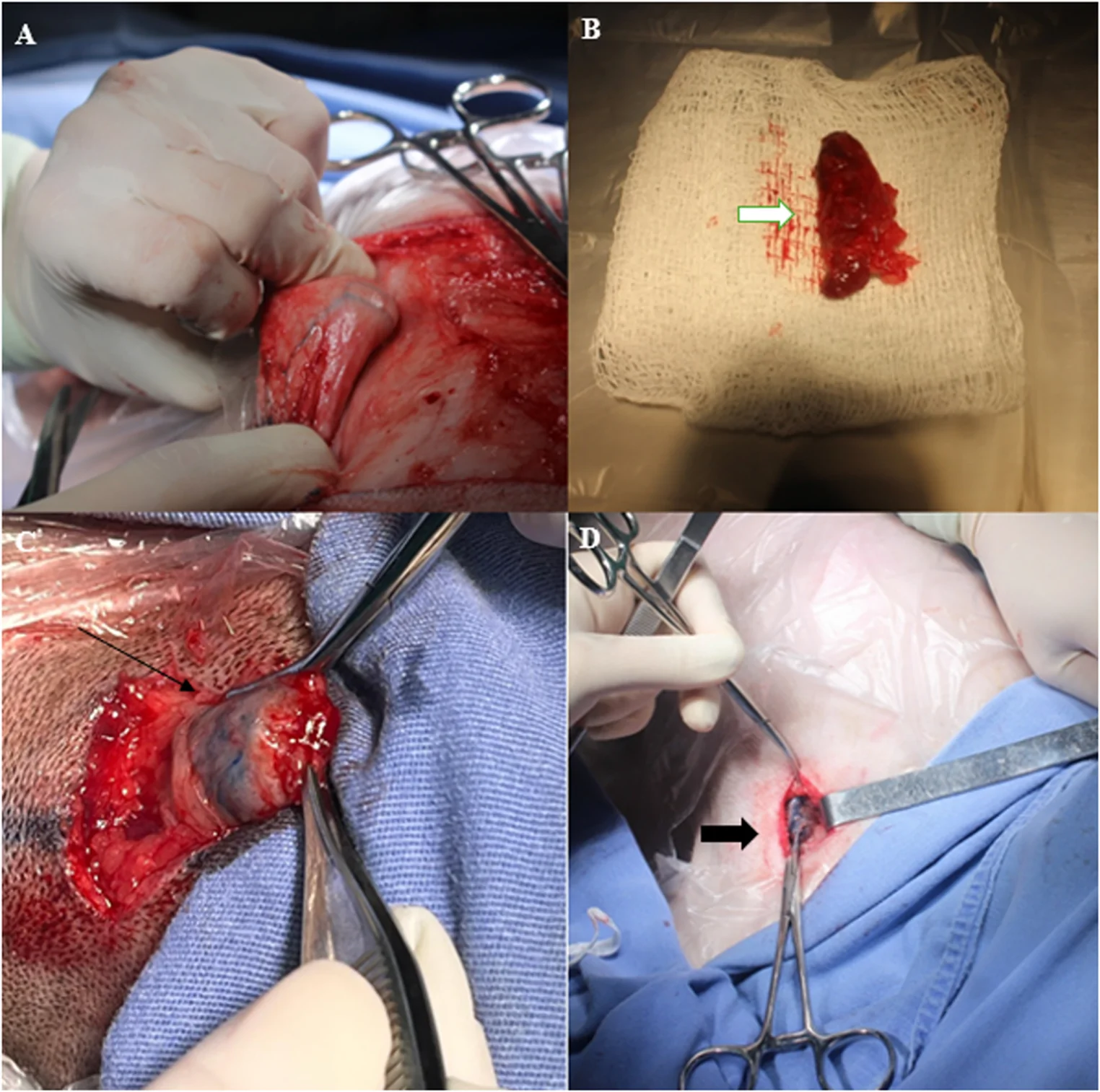
Lymphatic mapping with metilene blue in dogs. (A) Visualization of lymphatic vessels stained with blue dye. (B) White arrow: unstained sentinel lymph node. (C) Arrow: faintly stained sentinel lymph node. (D) Black arrow: intensely stained sentinel lymph node

In two tumors (7.7%), no SLN was detected surgically. The number of SLNs per tumor ranged from 0 to 3, with a mean of 1.31 ± 0.74 and a median of 1. Among tumors with at least one SLN detected (*n* = 24), 16 (66.7%) had a single SLN, 6 (25.0%) had two SLNs, and 2 (8.3%) had three SLNs. In the five tumors in which no contrasted-enhanced SLN was identified with IL, the sentinel node identified intraoperatively using MB was consistently the axillary lymph node, totaling six LN (one dog had two axillary LN). Methylene blue highlighted the lymphatic vessels and SLNs, which showed variable staining intensity (Fig. 3).

The dogs were monitored for the first two weeks after the procedure. None showed blue discoloration of the urine or mucous membranes, and no adverse reactions to methylene blue were observed. 

### Surgical procedure

In the present study, 21 animals underwent surgical resection of the tumor and the corresponding LNs, resulting in the excision of 38 lymph nodes. In some cases (*n* = 4), none of the evaluated techniques (IL or MB) detected the LN. Two dogs (9.5%) showed lipiodol uptake in the medial iliac LNs; however, these LNs were not excised due to the owner’s refusal to allow additional intervention. In three cases, intraoperative electrochemotherapy was performed because adequate surgical margins could not be achieved. Two thoracic-limb wounds developed dehisced with necrosis and were managed by open wound management. No systemic adverse effects were observed. 

Surgical margins were evaluated in 19 of 21 dogs (13 intraoperatively and 6 postoperatively); two limb amputations were excluded. During surgery, 7 of 13 (53.8%; 95% CI, 25.1–80.8) showed incomplete margins. Tumors with incomplete margins were larger (median 4.0 cm) than those with complete margins (median 1.5 cm; Mann–Whitney U = 32.5, *p* = 0.018; *r* = 0.70). Among cases with documented margin width, incomplete margins occurred in 2 of 2 excisions with 2 cm margins and 5 of 11 (45%) with 3 cm margins (>p = 0.462), indicating limited statistical power. On postoperative histopathology, 5 of 6 cases had incomplete margins (four despite 3 cm margins); the only complete excision was observed in the smallest tumor (0.7 cm). Overall, larger tumors were more often incompletely excised, and no significant difference was observed between 2 and 3 cm margins in this small sample. 

A total of 38 lymph nodes were removed, and most of the excised SLN were located in the inguinal and axillary lymphocenters. Complications following lymphadenectomy occurred in five cases, most frequently in the superficial cervical region (Table 2). Two additional axillary LNs were excised based on clinical judgment rather than SLN mapping (no lipiodol uptake, no methylene-blue staining). Both were classified as HN1 on histopathology and were therefore not counted as SLNs; excluding them does not alter the study estimates. Complications at the primary tumor site occurred in five dogs, predominantly wound dehiscence.

**Table 2:** Incidence of lymph node complications in dogs with cutaneous mast cell tumor (*n* = 21 animals; 38 lymph nodes)

Sentinel lymphocenter	Total excised lymph node	Complication	Incidence
Superficial cervical	10	2	20%
Popliteal	3	1	33,3%
Inguinal	14	1	7,14%
Axillary/accessory axillary	11	1	9,1%
Total	38	5	-

### Agreement of IL and MB

A total of 26 tumors were evaluated in 21 animals. Indirect lymphography identified lymphatic drainage pathways and presumptive SLNs in 21 of 26 tumors (80.8%; 95% CI, 60.6–93.4). Based on these lymphographic findings, intraoperative MB-assisted exploration enabled surgical identification of SLNs in 24 of 26 tumors (92.3%; 95% CI, 74.9–99.1).

IL detected 29 SLNs, whereas MB identified 34 SLNs. A total of 21 SLNs were identified by both IL and MB. In five tumors in which IL failed to identify a SLN, MB subsequently identified six axillary SLNs, including two nodes in one dog. The corresponding primary tumors were located in the trunk (dog 1), lateral thorax (dog 3B), thorax (dog 14), near the axilla (dog 20), and cranial abdomen (dog 21). In all cases, the SLNs detected exclusively by MB were axillary lymph nodes. Although MB identified a greater number of metastatic nodes overall (HN2–HN3), both techniques showed a similar distribution across nodal histopathological grades. SLN metastasis (HN2–HN3) was identified in 12 of 21 dogs (57.1%). A total of 38 lymph nodes were analyzed, of which 18/38 (47.4%; 95% CI, 31.0–64.2) were metastatic (HN2 = 15; HN3 = 3). 

To evaluate the association between primary tumor size and the presence of lymph node metastasis, one tumor per dog was considered (for dogs with multiple tumors, the largest tumor was selected). Tumors were divided into three groups according to maximum diameter: ≤2 cm, > 2 to ≤ 3 cm, and > 3 cm. Metastatic involvement (HN2/HN3) occurred in 3/10 (30%) dogs with tumors ≤ 2 cm, 3/3 (100%) with tumors > 2 to ≤ 3 cm, and 6/8 (75%) with tumors > 3 cm. Fisher’s exact test showed a significant difference among groups (*p* = 0.048), indicating a positive association between tumor size and nodal metastasis (Table 3). Mean primary tumor size increased with higher nodal grades (HN0: 1.5 cm; HN1: 3.07 cm; HN2: 3.59 cm; HN3: 3.82 cm). Histologic grading was available for all tumors. Most were classified as Patnaik grade II/Kiupel low-grade (24/26), while two tumors were classified as high-grade. One of the high-grade tumors was associated with HN3 lymph node metastasis, whereas the other showed HN1 lymph node involvement. Because only two high-grade tumors were included in the study population, statistical analysis evaluating the association between histologic grade and nodal metastasis was not performed.

**Table 3:** Association between tumor size and lymph node metastasis (HN2/HN3) in dogs with cMCT

Tumor Size Group	Total Dogs	HN2/HN3 *n* (%)	HN0/HN1 *n* (%)
≤ 2 cm	10	3 (30)	7(70)
> 2 and ≤ 3 cm	3	3 (100)	0(0)
> 3 cm	8	6 (75)	2(25)
Total	21	12 (57.1)	9(42.9)

Concordance with the expected RLN (Suami et al. 2013) was 76.9% (20/26; 95% CI, 56.4–91.0) for MB and 61.5% (16/26; 95% CI, 40.6–79.8) for IL; when considering the union of both methods, RLN concordance increased to 84.6% (22/26). Unexpected lymphatic drainage patterns included drainage from pinna and elbow tumors to the superficial cervical lymph node rather than the anatomically predicted regional lymph node, dual drainage from a hindlimb tumor to the popliteal and inguinal lymph nodes, drainage from a peri-axillary tumor to the superficial cervical, axillary, and accessory axillary lymph nodes, and drainage from a radial tumor to both the superficial cervical and axillary lymph nodes. The two methods were concordant in 16/26 cases (61.5%) meaning they identified identical SLN sets. MB identified more SLNs overall than IL (34 vs. 29 across 26 tumors) and, in the axillary basin, mapped six SLNs in five dogs, whereas IL did not identify any. IL identified more than one SLN in 8 of 26 tumors (30.7%) (Table 4).

**Table 4:** Regional lymph node (Suami et al. 2013), tumor location, and sentinel lymph node (SLN) in dogs with cMCT

Dog#	Tumor location	Regional LN (Suami)	SLN – IL	SLN – MB
1	trunk	Axillary	none	Axillary (HN1)
2	pinna	Medial retropharyngeal	superficial cervical (2) (HN1)	superficial cervical (2) (HN1)
3a	hindlimb (digit)	Superficial inguinal	popliteal, right inguinal (2) (HN2)	popliteal, right inguinal (2) (HN2)
3b	lateral thorax	Axillary	none	Axillary(HN2)
4	forelimb (digit)	Superficial cervical	superficial cervical (2) (HN2)	superficial cervical (2) (HN2)
5	perivulvar	Medial iliac	right inguinal(HN3)	right inguinal (HN3)
6	hindlimb	Inguinal	Popliteal(HN2),inguinal (HN1)	Popliteal(HN2), inguinal (HN1)
7	abdomen	Inguinal	Inguinal (HN1) (2)	inguinal (HN1) (2)
8	elbow	Axillary	superficial cervical (HN1)	superficial cervical (HN1)
9	elbow	Axillary	superficial cervical (HN2)	superficial cervical (HN2)
10	radius	Superficial cervical	superficial cervical (HN2)	superficial cervical (HN2)
11	caudal abdomen	Inguinal	Inguinal (HN2)	none
12a	tarsus	Inguinal	Inguinal (HN0), popliteal (HN0)	Inguinal(HN0)
12b	lateral thorax	Axillary	Axillary (HN1)	Axillary (HN1)
13a	caudal abdomen	Inguinal	Inguinal (HN2)	Inguinal (HN2)
13b	caudal abdomen	Inguinal	Inguinal (HN2)	Inguinal (HN2)
14	thorax	Axillary	none	Axillary (HN0)
15a	near axilla	Axillary	superficial cervical(HN1)	superficial cervical (HN1), axillary(HN2), accessory axillary (HN1)
15b	left abdominal side	Inguinal	Inguinal (HN3)	Inguinal (HN3)
16a	right abdominal side	Inguinal	inguinal (2) (HN2)	inguinal (2) (HN2)
16b	neck	Superficial cervical	superficial cervical (HN1)	superficial cervical (HN1)
17	hindlimb	Inguinal	Inguinal (HN3)	none
18	radius	Superficial cervical	Axillary (HN0), superficial cervical(HN1)	superficial cervical (HN1)
19	near axilla	Axillary	axillary	Axillary (HN2)
20	near axilla	Axillary	none	Axillary (HN1)
21	cranial abdomen	Axillary	none	axillary (2) (HN1)
		**Totals (SLNs detected)**	**29**	**34**

Abbreviations: RLN = regional lymph node; SLN = sentinel lymph node

Descriptive frequency analysis revealed that both LN status and surgical margin pattern influenced chemotherapy recommendations, although in different ways. Among dogs with complete margins, 6 of 9 (66.7%) were recommended to receive adjuvant therapy, whereas this proportion increased to 9 of 10 (90.0%) among those with incomplete margins. Considering LN status, none of the animals classified as HN0 were recommended for chemotherapy, while the frequency progressively increased in the other groups: 57.1% in HN1, 71.4% in HN2, and 100% in HN3. Overall, margin status influenced recommendations, but LN involvement was the more consistent determinant of clinical management. 

## Discussion

The results of the present study support previous data regarding the epidemiology of cMCT. The mean age of 9.5 years was similar to the reported median of 8–9 years with cMCT. Another similarity was the anatomical distribution of tumors, with the trunk and limbs being the most affected regions. In our study these locations accounted for 42.3% (trunk) and 46.3% (limbs) of cases (Rodríguez et al. 2023). Mixed-breed dogs were the predominant group (52.4%), followed by Golden Retrievers (*n* = 2). This distribution is consistent with previous studies indicating that the mixed-breed dogs are among the most commonly cMCT-affected groups (Pierini et al. 2020; Stefanello et al. 2024), although the Golden Retriever have also been described as a breed predisposed to this neoplasm (Arendt et al. 2015; Biasoli et al. 2019). 

Tumor size showed a clear association with the likelihood of incomplete surgical margins. Larger tumors were significantly more often associated with incomplete margins, corroborating previous reports that identified tumor size as a major predictor of surgical outcome. Some studies suggest that the vast majority of grade I and II cMCTs can be completely excised when resected with a 2 cm lateral margin and one deep fascial plane (Fulcher et al. 2006). In addition, a modified proportional margins approach has been proposed for cMCT (Pratschke et al. 2013; Saunders et al. 2021). Because the optimal margin likely varies according to tumor size, location, histologic grade, and the need for planned adjuvant therapy, it remains uncertain; therefore, margin decisions should be individualized and supported by histopathology evaluation. 

Our results showed a significant association between larger tumor size and LN metastasis. Metastatic involvement was more frequent in larger tumors, and mean tumor size increased progressively with higher nodal grades (HN0: 1.5 cm; HN1: 3.07 cm; HN2: 3.59 cm; HN3: 3.82 cm). Similar findings were previously described (Ferrari et al. 2018) and observed that MCTs greater than 3 cm were frequently associated with metastatic LNs. From a clinical perspective, these findings reinforce the importance of careful LN evaluation in patients presenting with larger lesions, as they may warrant more aggressive staging strategies and closer postoperative monitoring. 

While lipiodol identified lymphatic drainage pathways and SLNs in 80.8% (21/26) of tumors, intraoperative MB- assisted exploshowed a higher detection rate, identifying SLNs in 92.3% (24/26). However, these techniques should not be interpreted as fully independent or directly comparable methods, since MB-guided surgical identification was performed based on the anatomical information provided by indirect lymphography. Similarly, a previous study reported a success rate of 89.6% for lipiodol in dogs with MCTs (De Bonis et al. 2022). However, a key limitation of that study was the absence of intraoperative confirmation of SLN identification, which may have influenced the reported outcomes. In contrast, in our study, both techniques were concordant in 73.1% (19/26) of cases, primarily involving superficial cervical and inguinal LN. 

Besides the moderate agreement between the two techniques, SLN metastasis (HN2-HN3) occurred in 12 of 21 dogs (57.1%), a proportion higher than that reported in previous studies, which described rates of approximately 50% (Ferrari et al. 2018; Stefanello et al. 2024). In previous studies, regional LNs were often evaluated based on proximity rather than true SLN mapping, although combined approaches have also been described, such as preoperatively lymphoscintigraphy followed by intraoperative MB injection guided by a gamma probe. Therefore, our protocol should be interpreted as a complementary mapping strategy, in which IL provided preoperative lymphatic mapping and MB facilitated intraoperative SLN visualization and surgical retrieval. Our combined IL and MB approach may improve the detection of clinically meaningful nodal metastasis compared with strategies based solely on removal of the expected regional LN. 

In our study, discordance between expected RLN and SLN occurred in 5 dogs, and 19% (5/26) of tumors had at least one additional SLN. This contrasts with previous findings reporting that up to 42% of dogs with cMCT may have an SLN different from the expected RLN (Worley 2014). 

MB facilitated intraoperative identification of axillary nodes in 5 tumors in which lipiodol failed to demonstrate nodal opacification. Conversely, lipiodol identified inguinal nodes in 2 of 26 tumors that were not detected by MB. These findings reinforce the complementary nature of the two approaches, as IL provided anatomical mapping of lymphatic drainage pathways, whereas MB improved real-time intraoperative visualization of SLNs and lymphatic vessels. Previous studies have suggested that MB can be used as a single technique, with staining observed in approximately 88% of dogs and a consistent association between stained LNs and metastatic involvement (Zanardi et al. 2025). However, IL revealed alternative or multiple lymphatic drainage pathways that were not detected by MB, which may influence the tumor staging. Thus, while MB is simple to use and widely accessible in settings with limited resources, combining both techniques appears to improve mapping accuracy by identifying both expected SLNs and additional lymphatic drainage pathways. Notably, in our study MB also stained non-metastatic nodes (HN0), which contrasts with previous reports in which only metastatic nodes (HN2/HN3) were stained, whereas unstained nodes were classified as non-metastatic (HN0/HN1)(Zanardi et al. 2025). 

In five tumors, the IL failed to opacify SLN. In three cases, the IL injection was repeated, whereas in the remaining two cases reinjection was not possible because postponing the surgical procedure was not considered appropriate. Similarly, one study reported failure to detect SLN on the initial attempt in some patients, requiring a second contrast injection (Fournier et al. 2021). Interestingly, all failures in our study involved axillary SLNs. This observation suggests that anatomical factors, such as complex thoracic lymphatic drainage, superimposition with bony structures, or local adipose tissue volume that may impair contrast diffusion or radiographic delineation. In these situations, computed tomography lymphography could potentially improve lymph node detection, especially in regions with complex anatomy or subtle contrast uptake (Rossi et al. 2018). However, its routine use may be limited in some clinical settings because of cost and availability. In addition, technical factors such as insufficient contrast volume or accidental subcutaneous rather than intradermal injection may also have limited nodal opacification. In these cases, all SLNs were identified using MB, and four were classified as HN1 (pre-metastatic). Thus, lack of opacification on IL did not exclude the presence of SLNs or clinically relevant nodal alterations. This finding is consistent with a previous study in which, in 17% of cases, SLNs were localized by tracking contrasted-enhanced lymphatic channels rather than by direct nodal uptake (Lapsley et al. 2021). Although metastatic obstruction of lymphatic flow can prevent nodal opacification (Soultani et al. 2017), this mechanism is more consistent with advanced disease and is therefore less likely to explain the predominantly HN1 findings observed in the present study. 

From a biological perspective, tumor biology likely plays a central role in these findings. In canine mast cell tumors, early lymphatic dissemination may occur before significant structural disruption of lymphatic vessels or lymph nodes becomes evident, allowing partial preservation of lymphatic drainage during the initial stages of disease (Krick et al. 2009; Weishaar et al. 2014). In addition, tumor-associated changes in lymphatic function and interstitial pressure may further influence lymphatic flow dynamics, even in the absence of overt metastasis (Mortimer and Rockson 2014). Patient-related factors may also contribute to mapping variability. In particular, increased adipose tissue may not only interfere mechanically with contrast diffusion and imaging but has also been associated with reduced SLN detection rates, possibly due to altered lymphatic function and increased interstitial resistance (Insalaco et al. 2024). 

Few adverse reactions were observed, occurring in three cases and characterized by a local response within 24 h after IL administration, marked by erythema and histopathological evidence of lymphoplasmacytic dermatitis. These changes resolved following surgical excision of the affected skin. Similarly, erythema and swelling at the injection site have been reported in up to 50% of cases, typically occurring 2–4 weeks after indirect lymphangiography (Mayer et al. 2013). Although the reactions observed in the present study occurred earlier and resolved without additional complications, both findings indicate that localized inflammatory responses may occur after lipiodol injection. This is likely related to its oil-based nature, which can lead to local tissue irritation, a mild foreign body–type reaction, and prolonged retention within lymphatic vessels, contributing to a transient inflammatory response (Lee et al. 2014; Sugiyama et al. 2024). However, these reactions are generally self-limiting, indicating that IL remains a safe technique. In contrast, no adverse effects were associated with MB in the present study. Only five animals developed mild postoperative complications, which seroma, lymphedema, and lameness. Seroma formation may be attributed to the accumulation of serous fluid within surgical dead space, resulting from disruption of lymphatic and vascular structures during tissue dissection, while lymphedema is likely associated with transient impairment of lymphatic drainage following lymph node removal. Lameness may be explained by local inflammation, tissue manipulation, or temporary discomfort related to the surgical procedure (Fossum 2019; Kuroi et al. 2006; Mortimer and Rockson 2014). These complications were transient and resolved within a few days after surgery, either spontaneously in most cases or with supportive corticosteroid therapy. The overall incidence of postoperative morbidity was 13.2% (5/38), which is consistent with previous studies reporting a low frequency of complications (21.2%) associated with lymphadenectomy (Chiti et al. 2023; Mattioli et al. 2025). The combination of lipiodol with MB facilitates the visualization of the LN in relation to the surrounding tissues, thereby reducing the extent of manipulation and anatomical dissection, as well as surgical morbidity for the patient, in agreement with the literature. Therefore, lymphadenectomy of peripheral LNs appears to be a safe technique that can be readily performed in most cases. 

The limitations of the study include the small sample size, variability in tumor locations, and the lack of randomization and blinding, which may limit the generalizability of the results. In addition, long-term follow-up and survival analysis were not available in the present study, preventing assessment of the impact of SLN mapping and lymphadenectomy on oncologic outcomes. Prospective multicenter studies with standardized protocols and follow-up are needed to further evaluate the clinical relevance of these techniques, including their effects on local control, disease-free interval, and overall survival. 

## Conclusion

Combining lipiodol-based indirect radiography and intraoperative MB staining offers a highly effective, complementary approach for identifying the SLN in dogs with cMCTs. 

Concordance with the expected regional LNs basin increased to 84.6% when both techniques were considered together, and 19% of tumors had additional SLN outside the expected regional basin. Importantly, lack of opacification with IL did not rule out clinically relevant SLNs, and MB staining was not perfectly specific for metastasis. Adverse effects were uncommon and mild, and postoperative morbidity after lymphadenectomy was low, supporting the safety and practicality of this approach. 

## Data Availability

No datasets were generated or analysed during the current study.
